# Health Care Quality in CKD Subjects: A Cross-Sectional In-Hospital Evaluation

**DOI:** 10.1155/2022/9432509

**Published:** 2022-06-23

**Authors:** L. Rzayeva, I. Matyukhin, O. Ritter, S. Patschan, D. Patschan

**Affiliations:** Zentrum Innere Medizin 1, Universitätsklinikum Brandenburg, Medizinische Hochschule Brandenburg, Brandenburg an der Havel, Germany

## Abstract

**Methods:**

The study was performed in a retrospective and observational manner. All adult (age 18 years or older) in-hospital subjects treated from January until December 2019 were included. CKD was diagnosed according to the KDIGO 2012 CKD Guideline. The following variables were assessed: CKD stage, quantification/analysis (yes/no) of blood pressure, proteinuria, serum phosphate, serum 25-OH-D3, ferritin and transferrin saturation, and blood gas analysis. In addition, recommendations of the following medicines were analyzed (given/not given): ACE inhibitor or sartan, phosphate binder, vitamin D3 (activated or native), iron, erythropoietin, and bicarbonate. It was also evaluated whether discharge letters contained CKD-related diagnoses or not.

**Results:**

In total, 581 individuals were included in the study. The majority of aspects related to the monitoring and therapeutic management of CKD were either considered in only a small proportion of affected individuals (e.g., quantification of PTH − 5.5%/25-OH-D3 − 6%/transferrin saturation − 13.6%) or avoided nearly at all (e.g., recommendation of erythropoietin—1%, documentation of CKD-MBD diagnosis—0.3%). A reasonable quality of care was identified concerning the blood pressure monitoring (performed in 100%) and blood gas analysis (55% of the patients received analysis). Serum phosphate was measured in 12.9%, particularly in subjects at higher CKD stages.

**Conclusions:**

The current investigation revealed poor quality of care in CKD patients treated at the Brandenburg University Hospital over the period of one year. Quality improvement must be achieved, most likely via a standardized educational program for physicians and a directer access to CKD management guidelines.

## 1. Introduction

Chronic kidney disease (CKD) [1] is an emerging problem in both clinical and ambulatory medicine. In Central Europe and the US, CKD prevalence has continuously increased in recent years, which particularly results from increasing global prevalence of diabetes mellitus [2, 3]. Meanwhile, the latter is the most common single cause of CKD [4]. It is estimated that 40% of the subjects with end-stage kidney disease (ESKD) suffer from diabetes. The prognosis of CKD patients critically depends on the prevalence and severity of cardiovascular (CV) complications, as emphasized by the landmark study of Go and colleagues [5] more than 15 years ago. The significant CV risk increase in CKD results from the accumulation of traditional [6] and nontraditional risk factors such as albuminuria, renal anemia, total CO2 content, alkaline phosphatase, and fibroblast growth factor-23 (FGF-23) [7, 8]. New CKD therapeutics have been identified in recent years (gliflozines and finerenone [9–12]). Gliflozines, for instance, has been shown to slow down the GFR loss over time, independently of the glucose metabolism [10]. In 2021, Dapagliflozin was approved for CKD subjects with and without preexisting diabetes mellitus [13]. Nevertheless, much effort in terms of managing CKD must be put into the control of so-called progression factors. In this regard, distinct recommendations have been provided by the “Kidney Disease Improving Global Outcomes” (KDIGO) initiative [14]. A prerequisite for adequate control of CKD progression is regular monitoring of numerous clinical and laboratory surrogates. In this regard, established guidelines are being updated regularly [14, 15].

In the current investigation, we evaluated the CKD-associated health care quality in all in-hospital subjects that were treated during the year 2019. The study focused on quality of blood pressure and proteinuria control, renal anemia, bone metabolism, and renal acidosis.

## 2. Methods

### 2.1. Setting

In 2014, the Brandenburg Medical School was founded as the first university of medicine in the whole federal state of Brandenburg (Germany). Its fundamental mission is to improve the quality of health care in rural communities. In summer 2017, a new department of nephrology was opened as part of the University Hospital Brandenburg. It was the first time in more than 20 years that a nephrologist became responsible for managing patients with various types of kidney disease. Currently, the Brandenburg university hospital is the only hospital localized in the city of Brandenburg. Patients with kidney disease receive in-hospital treatment if necessary, and extracorporeal therapies are performed on a dialysis unit if mandatory. In addition, an outpatient unit provides health care for ambulatory patients. Besides the university hospital, only one additional outpatient unit provides diagnostics and treatment for subjects with kidney disease. Most patients receive health care coverage from statutory health insurance companies.

### 2.2. Patients

The study was retrospective and observational. The ethics committee of the Medical School of Brandenburg approved the study (No. E-02-20200602). It was not mandatory to obtain written consent to participate due to the retrospective and observational character of the investigation. All patients treated at the University Hospital Brandenburg from January until the end of December 2019 were screened. Data were extracted from the central database of the hospital (MEDICO® by CGM). All patient's histories and treatment-associated clinical data are stored in the database. The same applies to all laboratory findings and the medication prescribed before hospital admission and then after. Information about follow-up recommendations was extracted from individual discharge letters.

### 2.3. CKD Diagnosis

Two inclusion criteria were defined: (I) age of at least 18 years or older and (II) the diagnosis of CKD was according to the KDIGO 2012 CKD Guideline [14]: (I) reduction of the estimated glomerular filtration rate (eGFR) to under 60 mL/min, calculated with the CKD-EPI formula [16] and/or (II) an urine albumin-to-creatinine ratio (ACR) of >30 mg/g (random urine sample) and/or (III) structural abnormalities of the kidney(s), revealed by ultrasound analysis. Since information about proteinuria and kidney ultrasound findings were not available in many subjects, the diagnosis “CKD” was exclusively made if the eGFR to under 60 mL/min for longer than 3 months. Not included were patients with acute severe diseases such as sepsis or septic shock or patients with the uncontrolled malignant disorder. The time frame of 3 months was an essential prerequisite for study inclusion. Subjects with CKD stages V ND (no dialysis) and V D (dialysis) were summarized in the category stage V.

### 2.4. Variables of CKD Health Care Quality

Three general quality categories were defined as follows: (I) blood testing for CKD-related surrogate markers, (II) recommendation of antiprogressive medications, and (III) documentation of CKD-related diagnoses in the discharge letter. Quality category I: quantification/analysis (yes/no) of proteinuria, serum phosphate, serum 25-OH-D3, ferritin, and transferrin saturation, and blood gas analysis. Quality category II: recommendations of one or more of the following substances (yes/no): ACE inhibitor or angiotensin II type 1 receptor blocker (termed as “sartan(s)” throughout the article), phosphate binder, vitamin D3 (activated or native), iron, erythropoietin, and oral bicarbonate. Quality category III: documentation of one or more of the following diagnoses (yes/no): CKD, CKD stage, arterial hypertension, “CKD-MBD” or “renal osteopathy” or “renal osteodystrophia,” renal anemia, and renal acidosis. The respective CKD stage was defined according to KDIGO [14], based on the eGFR calculated with the CKD-EPI formula [16]. Systolic and diastolic blood pressures before discharge were assessed also. The diagnosis of arterial hypertension was either made if indicated by the patient's history and medication or if more than 25% of all documented blood pressure values during the in-hospital treatment were above 140/90 mmHg.

### 2.5. Statistics

Before any analysis, all data were checked for normality using the Kolmogorov–Smirnov test. Two groups were compared with the Student's *t*-test if normality was fulfilled or with the Mann–Whitney test if the results were not distributed normally. Three or more groups were compared with ANOVA if the data were distributed normally or with the Kruskal–Wallis test if the normal distribution was not given. Results are either given as percentages or as mean ± SEM. Statistical significance was postulated if the *p* value was below 0.05. All analyses were performed with the following application: Wizard® for Mac OS (version 2.0.10, developer Evan Miller).

## 3. Results

### 3.1. Patients

**Figure 1:**
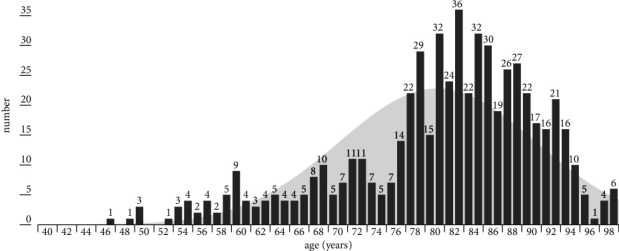
Age distribution of all included patients.

In total, 581 individuals were included in the study (females 309, males 272). The mean age was 81.2±10.1 years (82.3±0.6 years in females; 80±0.5 years in males). The age distribution is shown in Figure 1. The mean time of in-hospital treatment was 13.1±10.8 days (all), 12.6±0.6 days (females), and 13.5±0.6 days (males). The distribution of the CKD stages II, IIIa, IIIb, IV, and V was 2.4, 12.7, 35.1, 39.6, and 10.2 (%), respectively. All patients' characteristics are summarized in Table 1.

### 3.2. Arterial Hypertension

**Figure 2:**
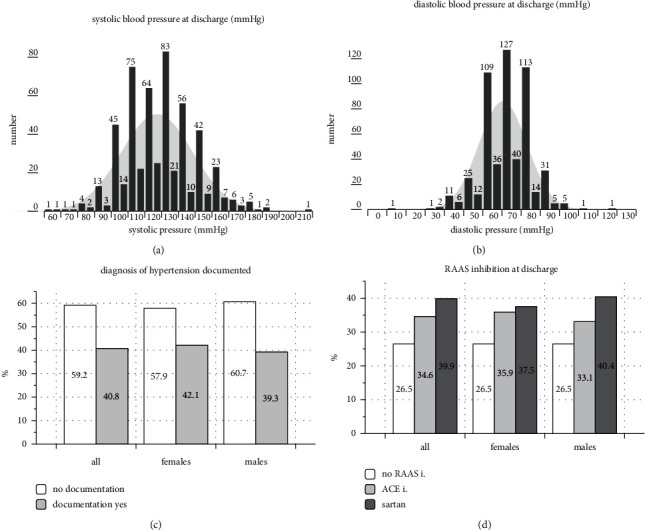
Systolic/diastolic blood pressure and RAAS inhibitor recommendation at the time of discharge. (a) Systolic blood pressure results (mean 127±21 mmHg); (b) diastolic blood pressure results (mean 70±12 mmHg); (c) documentation of the diagnosis of hypertension at discharge; (d) RAAS inhibitor recommendation at the time of discharge (abbreviations: RAAS i, RAAS inhibition; ACE i, ACE inhibitor).

The mean systolic blood pressure at the time of discharge was 127±21 mmHg, and the diastolic pressure was 70±12 mmHg. On the day of discharge, blood pressure was evaluated in all subjects. The diagnosis of hypertension was documented in 59.2% of all discharge letters. A recommendation for RAAS inhibition was not given at all in 26.5%. An ACE inhibitor was recommended in 38.9%, and an angiotensin II inhibitor was recommended in 34.6% (Figure 2).

### 3.3. CKD-MBD

**Figure 3:**
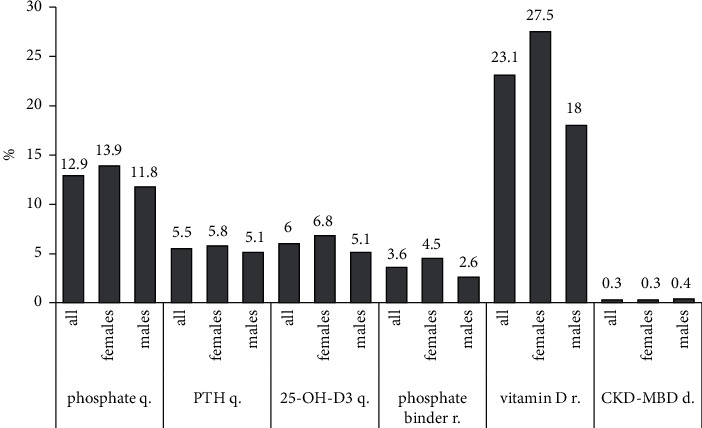
CKD-MBD management. It illustrates whether serum phosphate, PTH, and 25-OH-D3 were quantified during in-hospital treatment and whether phosphate binders and vitamin D treatment were recommended at discharge (%). The last bar group shows the percentage of all discharge letters that contained a CKD-MBD-related diagnosis (abbreviations: d, documentation; q, quantification; r, recommendation).

It was analyzed whether the following parameters of bone metabolism were measured at least once during the hospital stay: serum phosphate, serum parathormone (PTH), and serum 25-OH-D3. Also, it was evaluated whether the discharge letter contained recommendations for the administration of phosphate binders and/or any vitamin D preparation and whether a bone-related diagnosis (CKD-MBD or renal osteodystophia/osteopathy) was listed. Serum quantifications were performed in the following percentages of all subjects: phosphate 12.9, PTH 5.5, and 25-OH-D3 6. Phosphate binder administration was recommended in 3.6%, and the usage of any vitamin D preparation in 23.1%. A bone-related diagnosis was listed in 0.3% (Figure 3).

### 3.4. Renal Anemia

**Figure 4:**
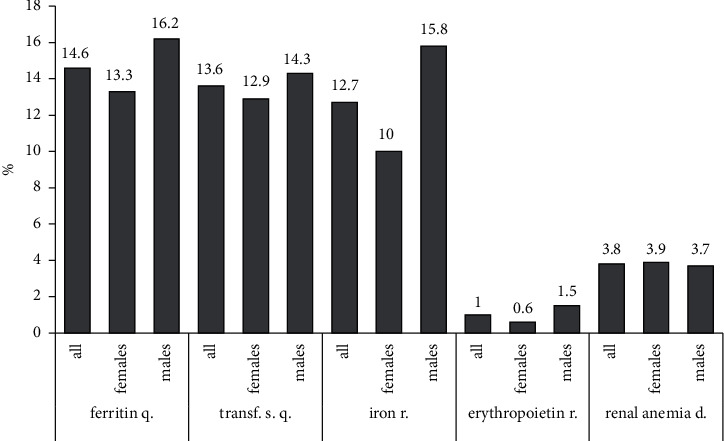
Management of renal anemia. It depicts the following categories: quantification of serum ferritin and transferrin saturation, recommendation of iron and erythropoietin therapy, and final documentation of renal anemia (discharge letter) (abbreviations: d, documentation; q, quantification; r, recommendation; s, saturation; transf, transferrin).

Among the parameters for monitoring renal anemia, our study evaluated serum ferritin and transferrin saturation. Serum ferritin was measured in 14.6%, and the transferrin saturation in 13.6%. Recommendations for iron and erythropoietin supplementation were given in 12.7% and 1%. Finally, the diagnosis of renal anemia was documented in 3.8% (Figure 4).

### 3.5. Proteinuria and Acidosis

**Figure 5:**
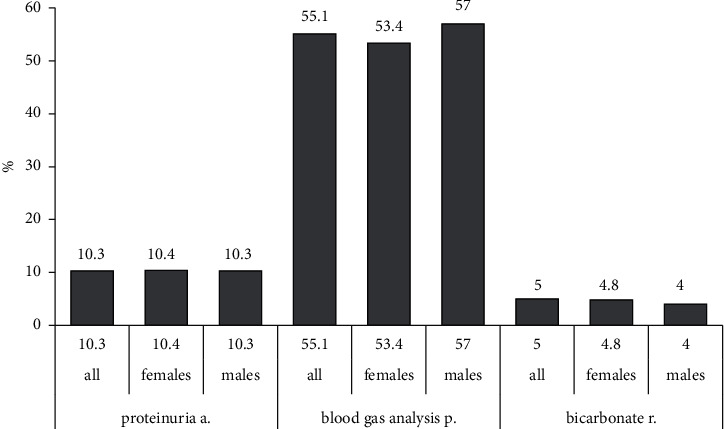
Management of proteinuria and acidosis. Three categories were evaluated: assessment of proteinuria and blood gas analysis during in-hospital treatment, and the recommendation of bicarbonate therapy at discharge (abbreviations: a, assessment; p, performed; r, recommendation).

Proteinuria was assessed in only 10.3% of all subjects. (Venous) blood gas analysis was performed in at least 55.1% of the patients, and a recommendation for regular bicarbonate supplementation was given in 5% (Figure 5).

### 3.6. Health Care Quality in CKD Stages II–V

**Figure 6:**
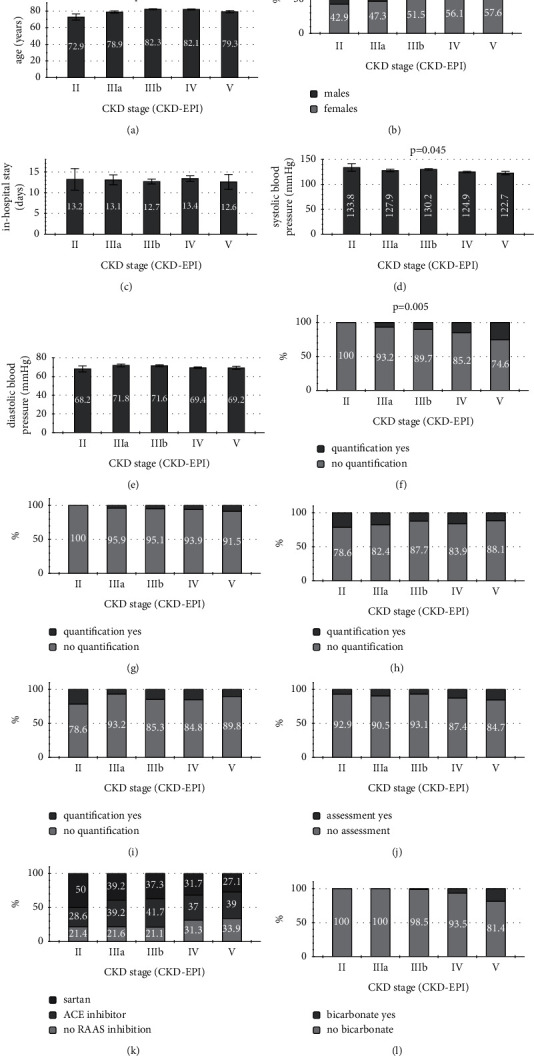
Distribution of certain variables in the CKD stages. (a) Age was unequally distributed in the stages II–V, the same applied for systolic blood pressure before discharge (d). (f) Serum phosphate quantification was performed more frequently in subjects with higher CKD stages (results in (a), (c), (d), and (e) as mean±SD). (a) Age, (b) gender, (c) in-hospital stay, (d) systolic blood pressure, (e) diastolic blood pressure, (f) phosphate quantification, (g) PTH quantification, (h) ferritin quantification, (i) quantification of transferrin saturation, (j) assessment of proteinuria, (k) RAAS inhibition, and (l) bicarbonate treatment.

Finally, the distribution of the following variables was analyzed in relation to the CKD stages II–V: age (years±SD), gender, duration of in-hospital stay (days±SD), systolic and diastolic blood pressure (mmHg±SD), quantification of serum phosphate, PTH, ferritin, transferrin saturation (all in %), assessment of proteinuria (%), RAAS inhibition (ACE inhibitor/sartan/no inhibition at all—%), and recommendation of oral bicarbonate (%). Three variables were distributed heterogeneously: age, systolic blood pressure, and quantification of serum phosphate. The latter was measured with increasing frequency from stage IIIa to V (IIIa: 6.8%; IIIb: 9.3%; IV: 14.8%; V: 25.4%; and *p*=0.005) (Figure 6).

## 4. Discussion

The current retrospective and observational investigation reveals a poor health care quality in CKD subjects, treated at an emerging university hospital for a period of one year. The majority of aspects related to the monitoring and therapeutic management of CKD were either considered in only a small proportion of affected individuals (e.g., quantification of PTH/25-OH-D3/transferrin saturation) or avoided at all (e.g., recommendation of erythropoietin and documentation of CKD-MBD diagnosis). Reasonable quality of care was identified concerning blood pressure monitoring, blood gas analysis (55% of the patients received analysis), and serum phosphate quantification. The latter, although performed in only 12.5% of all individuals, was at least initiated more frequently in higher CKD stages according to KDIGO.

In recent years, several studies reported on the quality of care for CKD. In 2019, Tummalapalli and colleagues [17] published a large-scaled cross-sectional investigation, in which more than 7,000 individual visits of CKD subjects were analyzed (period 2006–2014). The prevalence of uncontrolled hypertension and diabetes ranged from 40 to 48%, and the prevalence of ACE inhibitor use even decreased over time (45 to 36%). Another trial, published in 2020 [18] included >800,000 CKD patients, diagnosed with the disease between 2010 and 2014. Proteinuria was assessed in ∼60%, and only 4.5% received nutritional guidance. More than 90% avoided the use of nonsteroidal anti-inflammatory drugs. A Canadian study from 2019 [19] analyzed 46,162 CKD patients, extracted from the Care Sentinel Surveillance Network data (period 2010–2015). The authors defined 12 quality indicators, of which only 4 were met by at least 75% of the subjects. The rate of albuminuria assessment within 6 months of CKD diagnosis for instance was 18.4%. Thus, a lack of health care quality in CKD has been shown by others also. Regarding our study, several reasons may account for the poor quality of care. First of all, an in-hospital nephrologist was not in charge at all for more than 20 years. The sensitivity of physicians to kidney-related medical problems remains low until today, although a section of nephrology was opened in the summer of 2017. Second, the patients included were recruited from all subjects treated in any department of the hospital during the year 2019. These included general and trauma surgery, obstetrics, ear nose throat medicine, and others. What are the options for improving the quality of care for CKD? In 2019, Vassaloti and colleagues [20] reported a respective initiative. The proposed measures or interventions were part of the so-called “CareFirst's patient-centered medical home (PCMH) model,” which was developed in 2011. Its goal was to control the rising health care costs in Maryland (Virginia, US). Local care coordinators (LCCs) worked closely with primary care physicians (PCPs) to promptly identify subjects at risk for or with established CKD. The PCPs decided on necessary procedures for every individual (e.g., referral to a nephrologist). Initially, all PCPs underwent specialized education to ensure/increase the quality of care. The final population included more than 7,000 individuals. Interestingly, the interventions did not significantly increase the rates of ACE inhibitor or angiotensin receptor blocker use in hypertensive subjects with high-grade albuminuria. The authors, however, found reductions in-hospital admissions and 30-day readmissions per 1,000 patients, respectively. The study at least showed the feasibility of CKD care quality interventions. The quality improvement, however, was substantially achieved by increasing the awareness and knowledge of care coordinators and physicians. Thus, the key element for improving quality was education. Comparable observations were reported by Xu et al. [21], although derived from a study in patients with AKI (Acute Kidney injury). They established a multifaceted educational program, including didactic lectures, case-based teaching in small groups, and an interactive learning component. These measures significantly increased the percentage of physicians able to satisfactorily initiate adequate therapy upon the diagnosis of AKI. Once again, the key to quality improvement was education. A lack of physicians' expertise in the same field (AKI) was also reported in a 2020 published cross-sectional survey [22]. Only 5% out of 169 physicians from Omdurman Military Hospital had so-called good AKI-related practice. Finally, Adejumo et al. [23] found only 1.2% out of 82 physicians have good knowledge of AKI. In order to improve CKD-related health care at the Brandenburg University Hospital, a standardized educational program for physicians from all departments is indispensable. Additional tools may be considered, such as computer-based solutions. Regarding AKI, respective tools have meanwhile been implemented in many hospitals (AKI alert systems) [24]. Drawz and colleagues [25] emphasized the feasibility of electronic health records in the setting of CKD since the diagnosis is critically based on laboratory findings.

The current study has limitations. On one hand, we were not able to reliably identify subjects that were in definite need of receiving a certain type of medicine. The reason is, at least in part, the limited accessibility to individual patients via the central database. Another reason is the retrospective nature of the study. Prospective study designs usually ensure the documentation of more complete data sets. Finally, we did not apply standardized tools for assessing the health care quality but exclusively documented frequencies of diagnostic and therapeutic measures related to the diagnosis of CKD. In summary, the current retrospective investigation nevertheless revealed a poor quality of care in CKD patients treated at the Brandenburg University Hospital for the period of one year. Quality improvement must be achieved, most likely via a standardized educational program for physicians.

## Figures and Tables

**Table 1:** Patients' characteristics.

Variable		Results			*p* value, females vs. males
		All	Females	Males	
Gender		581	309 (53.2%)	272 (46.8%)	n.a.
Age (years±SD)		81.2±10.1	82.3±0.6	80±0.5	**0.006**
In-hospital stay (days±SD)		13.1±10.8	12.6±0.6	13.5±0.6	0.31
CKD stage (% of all individuals)		**Females: II—1.9, IIIa—14.3, IIIb—36.4, IV—37.1, V—9.2**			n.a.
		**Males: II—2.9, IIIa—12.7, IIIb—35.1, IV—39.6, V—10.2**			
CKD diagnosis documented (%)		90.0	89.3	92.6	0.16
CKD etiology documented (%)		82.1	79.9	84.6	0.14
Diagnosis of hypertension documented (%)		59.2	57.9	60.7	0.5
RAS inhibitor at the time of discharge (no+/−ACE inhibitor—sartan in %)	No	26.5	26.5	26.5	0.72
	ACE i	38.9	37.5	40.4	
	sartan	34.6	35.9	33.1	
Quantification of serum phosphate (%)		12.9	13.9	11.8	0.4
Quantification of PTH (%)		5.5	5.8	5.1	0.72
Quantification of 25-OH-D3 (%)		6	6.8	5.1	0.4
Phosphate binder recommended (%)		3.6	4.5	2.6	0.2
Vitamin D recommended (%)		23.1	27.5	18	**0.007**
Diagnosis of CKD-MBD documented (%)		0.3	0.3	0.4	0.92
Quantification of ferritin (%)		14.6	13.3	16.2	0.32
Quantification of transferrin saturation (%)		13.6	12.9	14.3	0.62
Iron therapy recommended if necessary (%)		12.7	10	15.8	0.03
Erythropoietin therapy recommended if necessary (%)		1	0.6	1.5	0.32
Diagnosis of renal anemia documented (%)		3.8	3.9	3.7	0.89
Assessment of proteinuria (%)		10.3	10.4	10.3	**0.98**
Blood gas analysis performed (%)		55.1	53.4	57	0.38
Bicarbonate therapy recommended (%)		5	4.8	4	0.32
Nephrology follow-up recommended (%)		27	25.6	28.7	0.4

Abbreviations: F, females; M, males; ACE i, ACE inhibitor.

## Data Availability

All data are available from the corresponding author upon reasonable request (daniel.patschan@mhb-fontane.de).
